# How are personality trait and profile agreement related?

**DOI:** 10.3389/fpsyg.2015.00785

**Published:** 2015-06-09

**Authors:** Jüri Allik, Peter Borkenau, Martina Hřebíčková, Peter Kuppens, Anu Realo

**Affiliations:** ^1^Department of Psychology, University of TartuTartu, Estonia; ^2^Estonian Academy of SciencesTallinn, Estonia; ^3^Cognitive Psychology Unit, Department of PsychologyLeiden, Netherlands; ^4^Department of Psychology, Martin-Luther-Universität Halle-WittenbergHalle, Germany; ^5^Institute of Psychology, Academy of Sciences of the Czech RepublicBrno, Czech Republic; ^6^Department of Psychology, Katholieke Universiteit LeuvenLeuven, Belgium

**Keywords:** self-other agreement, trait-centered approach, variable-centered approach, Asendorpf's index, Rank Consistency Index

## Abstract

It is argued that if we compute self-other agreement on some personality traits then we possess no or very little information about the individuals who are the targets of this judgment. This idea is largely based on two separate ways of computing self-other agreement: trait agreement (*r_T_*) and profile agreement (*r_P_*), which are typically associated with two different trait-centered and person-centered approaches in personality research. Personality traits of 4115 targets from Czech, Belgian, Estonian, and German samples were rated by themselves and knowledgeable informants. We demonstrate that trait agreement can be partialled into individual contributions so that it is possible to show how much each individual pair of judges contributes to agreement on a particular trait. Similarly, it is possible to decompose agreement between two personality profiles into the individual contributions of traits from which these profiles are assembled. If normativeness is separated from distinctiveness of personality scores and individual profiles are ipsatized, then mean profile agreement r_P_ becomes identical to mean trait agreement *r*_*T*_. The views that trait-by-trait analysis does not provide information regarding accuracy level of a particular pair of judges and profile analysis does not permit assessment of the relative contributions of traits to overall accuracy are not supported.

## Introduction

Personality judgments may reflect not only the traits by which a specific target individual can be distinguished from other targets but also stereotypes, biases, and method-specific variance (Cronbach and Gleser, 1953; Cronbach, 1955). Since between-rater agreement on personality trait ratings allows us to separate some of the different components of ratings, the use of multiple informants has become one of the most valuable tools in personality research (McCrae, 1994; Funder, 1999; Kenny et al., 2006; Vazire, 2006; Borkenau and Zaltauskas, 2009; Kandler et al., 2010; De Los Reyes et al., 2013). It has even been claimed that other-ratings are a more valid source of information than self-ratings when it comes to the relationship between personality traits and some external validity criteria (Kolar et al., 1996; Connelly and Ones, 2010). In any case, agreement between judges is a precondition for a property to really exist. For instance, if two judges disagree about the judged trait, they cannot both be right. At least one of them is wrong and it is likely that they both are mistaken (Funder, 1999). Thus, agreement is necessary for the accuracy of judgments of personality traits.

### Two principal ways to compute agreement

One problem in personality research is that there are two principal ways of computing agreement between the judgments of multiple informants. Typically, data are collected concerning *N* targets who are rated, in addition to themselves, by one or several judges on exactly the same set of *K* personality traits. Agreement is calculated as a correlation between pairs of informants, the self and a knowledgeable other, in their rank-order position on each individual trait (Biesanz and West, 2000). Thus, *trait agreement* (*r*_*T*_) is computed separately for each of *K* personality traits across all *N* pairs of judges. Although researches may be interested in a single trait (*K* = 1), the unit of the analysis is still traits rather than individuals. This is why it is called the variable-centered (trait-by-trait) approach (Magnusson and Torestäd, 1993; Bergman and Trost, 2006; Furr, 2009).

Another approach concentrates on a specific individual who, besides her- or himself, is also judged by one or several judges. This method correlates ratings of two judges of a given target across *K* personality traits. Since the focus of this approach is the similarity between two (or sometimes more) trait profiles, this form of agreement is called *profile agreement* (*r_P_*). Because agreement is calculated across *K* personality traits for each individual target-judge pair, it is often called the person-centered (person-by-person) approach (Bernieri et al., 1994; Funder, 1997, 1999; Bergman and Trost, 2006).

Generally, for most personality traits, informants tend to achieve at least moderate cross-observer agreement (Watson et al., 2000; Connolly et al., 2007; Connelly and Ones, 2010; Kenny and West, 2010). For example, the median cross-observer trait agreement in a number of studies using measures of the Five-Factor model was 0.40 or higher on all the Big Five personality dimensions (McCrae et al., 2004). Almost identical levels of self-other agreement were found in North American and cross-cultural samples (McCrae et al., 2004). The mean profile agreement across all target-informant pairs has been shown to be in the same range or even higher than for trait agreement (Pelham, 1993; Kenny and Winquist, 2001; McCrae, 2008; Borkenau and Zaltauskas, 2009; Allik et al., 2010b; Dobewall et al., 2013). However, there is no good explanation why both trait and profile agreement are often around 0.40, with profile agreement having slightly higher values. In this study, we suggest an answer to this question.

The two methods of computing agreement—trait and profile agreement—both have their advantages and shortcomings (e.g., Bernieri et al., 1994). Trait agreement may be more popular among researchers, mainly because trait correlation is the basis of factorial models of personality, irrespective whether we talk about the Five-Factor Model (FFM, McCrae and Costa, 1987; Goldberg, 1993; Allik et al., 2013) or any other of a number of factor models (Ashton et al., 2004; Lee and Ashton, 2008; Ashton and Lee, 2010). Analysis of covariation between personality traits is the main method for revealing the genetic, environmental, and error-related structure of personality measures (McCrae et al., 2001). However, according to some researchers, personality trait covariation models, such as the FFM, provide information that holds true only at the level of groups or populations, and may not provide any useful information about individuals (Borsboom, 2005). For example, it was claimed that, if a latent factor model fits a given population, it does not necessarily fit each or even any individual in that population (Borsboom et al., 2003; Molenaar and Campbell, 2009). Bernieri and colleagues expressed a similar concern: “The trait-by-trait analysis allows researchers to determine which traits are more accurately perceived by judges but does not lend easily to questions regarding the accuracy level of a particular judge” (Bernieri et al., 1994, p. 370). Thus, it is a dominant belief that trait correlation does not provide much information about individuals.

The study of self-informant profile congruence was once a prolific area of research (Bruner and Tagiuri, 1954; Taft, 1955) until Cronbach (Cronbach, 1955; Gage and Cronbach, 1955) published a series of papers in which it was suggested that various response components, such as “stereotype accuracy,” “elevation,” and “differential elevation” should be differentiated and taken into account. An unexpected effect of Cronbach's critique was the abolishing of nearly all research on self-other congruence for several decades (Funder and Colvin, 1997). This was an unusual turn of events, as Funder and Colvin keenly observed, where the scientific community simply panicked instead of adopting basic techniques to separate, for example, the distinctiveness of personality profiles (differential accuracy) from normativeness or stereotypes (Gage and Cronbach, 1955; Bernieri et al., 1994; Furr, 2008; Borkenau and Zaltauskas, 2009). Although profile agreement is considerably more subject to distortions than trait agreement, there are relatively simple ways to ameliorate the situation. Many of these techniques were suggested in papers with dramatic consequences (Cronbach and Gleser, 1953; Cronbach, 1955; Gage and Cronbach, 1955). Unlike trait-by-trait analysis, profile analysis generates an accuracy score for each target-informant pair. At the same time, profile analysis, as is typically believed, “does not permit us to assess the relative contributions of traits (or items) to overall accuracy” (Bernieri et al., 1994, p. 370).

Nevertheless, profile analysis has a clear advantage over calculating agreement within traits—that is, its statistical power (Borkenau and Zaltauskas, 2009). For example, the statistical power required to establish a correlation between two traits in a sample of 300 participants is equal to the power required to establish this correlation across 30 traits among just 11 participants (Borkenau and Zaltauskas, 2009). Obviously, such a gain in power is highly desirable.

Although trait and profile agreement are both legitimate ways to estimate self-other congruence, there is very little empirical or theoretical information about how these two forms of agreement are related to one another. Common sense, but not so many empirical studies, would suggest that trait- and profile-agreement have something in common. Usually, the mean values of both, as already mentioned, are in the same range (0.40 or higher). On the other hand, there is plenty of evidence that these two forms of agreement have substantively different interpretations, implying that they are not interchangeable (Bernieri et al., 1994; Kenny and Winquist, 2001; Connelly and Ones, 2010). The view that trait and profile agreement may have no substantial overlap is also strengthened by easily constructed examples of at least apparent disassociation between these two forms of agreement. It is easy to envision an artificial example where a correlation between two personality profiles is zero. For instance, although it is rather unlikely that one would obtain equal scores on all personality traits, it is still possible and perfectly compatible with many personality models (Allik et al., 2012). Should this happen, there would be no variance within these personality profiles and, as a result, self-other agreement is zero. At the same time, targets and their informants may report very similar or even identical levels on these personality traits, resulting in high trait correlations. Conversely, it is possible to imagine trait correlations which are only insignificantly different from zero, based on a considerable number of dyads who report similar profiles. In conclusion, these two—person-centered and variable-centered—approaches have sometimes revealed similar, and sometimes dissimilar, findings (Furr, 2009, p. 203).

### The present study

The aim of the present study is to examine the relationship between trait and profile agreement and, by doing so, to extend the existing literature on the topic in a substantial way. The starting point is the observation that both trait and profile agreement are computed from precisely the same data (Furr, 2009, p. 203). Analyzing cross-situational behavioral contingencies (the same set of traits was measured repeatedly), Furr noticed that a profile approach can be combined with a more traditional trait-centered approach (Furr, 2009). In particular, he demonstrated that, in terms of covariation, one form of agreement is directly related to another by sharing some common components of covariation (Furr, 2009, Appendix A in Supplementary Material). In this study, we followed this approach and noticed that formulas for the trait *r_T_* and profile correlation *r_P_* (see Appendix A in Supplementary Material) contain the same member—the product of self- and other-rated scores—which makes these two correlations inevitably dependent on each other. This dependence between trait and profile correlations becomes obvious when Pearson correlations are computed as means of the products of standard scores. We also demonstrate in the Appendix A in Supplementary Material that standardization of personality scores makes the difference between mean trait agreement *r*_*T*_ (averaged across all traits) and mean profile agreement *r*_*P*_ (averaged across all self-other dyads) predictably smaller. For example, if personality scores are double standardized by setting all trait means and all person means to zero, and all trait and person standard deviations to one, the mean trait *r*_*T*_ and the mean profile *r*_*P*_ agreement become identical. Thus, if we eliminate all differences between traits and profiles caused by elevation or scatter, then it does not matter whether we compute the mean trait or profile agreement. Consequently, for double-standardized *z*-scores, there is only one measure of average self-other agreement, irrespective of whether we start from traits or from individual profiles (see Appendix B in Supplementary Material for an example).

Another way to analyze relationships between trait and profile correlations is to decompose self-other agreement on traits into individual pairs' contributions to this correlation (Asendorpf, 1992; Allik et al., 2010b). Asendorpf (1990, 1992) proposed a simple mathematical method for partialling the Pearson product moment correlation between two variables into the contributions of the individual dyads to this overall correlation. The proposed coefficient of individual consistency *I_XY_*, which characterizes each individual *XY* pair, was defined so that their mean value across all pairs was identical with the correlation at the aggregate level (Asendorpf, 1990). Using certain well-known properties of *z*-transformed variables, the index of individual consistency can be expressed as a linear function of the squared differences of the *z*-scores of the two variables: *I_XY_* = 1 - (*z_X_* - *z_Y_*)^2^/2, where *z_X_* and *z_Y_* are *z*-scores of the two paired variables *X* and *Y* (Asendorpf, 1990).

This index articulates the transparent idea that dyads whose members occupy approximately the same position in their respective rankings contribute strongly to the overall correlation, whereas members of dyads who occupy very different ranking positions contribute less, or even contribute negatively, to the overall correlation. Evidently, the disparity between individual rankings of dyad members is inversely proportional to the overall correlation. However, something that would limit the applicability of this idea is if variables *X* and *Y* do not perfectly meet the requirements for correlation as a measure of stochastic relation: For example, they deviate from the normal distribution by being strongly skewed or distorted in some way or another. Usually, particular transformations (e.g., Fisher's *z*-transformation) are recommended to restore normality of distributions.

Another possibility is to devise a new index of individual consistency which relies on ranking information only. For example, Spearman's rank correlation *ρ* (rho) measures statistical dependence between two paired variables *X* and *Y* based on their ranks. As a direct analogy with Asendorpf's individual index of consistency *I_XY_*, we can propose a non-parametric *Rank Consistency Index* (*RnkCI*) *ρ_XY_* = 1 − 6·(*r_X_* - *r_Y_*)^2^/(*N*^2^ − 1), where *r_X_* and *r_Y_* are the ranks of the respective values on variables *X* and *Y* and *N* is the number of pairs. It is important to note that the mean value of *ρ_XY_* across all *N* pairs is identical to the Spearman rank correlation *ρ* at the aggregate level. Thus, *ρ_XY_* demonstrates how strongly each individual pair contributes to the overall correlation of some trait. If we average *ρ_XY_* across all *K* personality traits, then we can find the mean *RnkCI* ρ_*XY*_ which characterizes how consistent ranks of self- and other-ratings across all *K* personality traits are. One obvious advantage of *RnkCI* is that it is based on much less restrictive assumptions than the Pearson product-moment correlation. The only underlying assumption of *RnkCI* is preservation of a monotonic relation.

Knowing the mean *RnkCI* (ρ_XY_) characterizing each individual dyad, we can compare it with the profile correlation *r_P_* computed across *K* traits for individual pairs. Profile agreement(r_P_) starts from individual target-judge pairs, measuring agreement between their two profiles. Obviously, *r_P_* can be averaged across multiple target-judge pairs, resulting in r_P_. To the best of our knowledge, so far nobody has compared ρ_XY_ and r_P_. If these two indices (r_P_ and ρ_XY_) are related to each other, this would disconfirm the view that person-centered and trait-centered analyses result in radically different findings on self-other agreement.

Some researchers have criticized the fact that results from the majority of psychological research have little relevance to the majority of the world (Berry et al., 2002), since they study predominantly WEIRD (Western, Educated, Industrialized, Rich, and Democratic) people (Henrich et al., 2010a,b; Jones, 2010). Indeed, a recent survey of the top psychological journals found that 96% of all research participants were from Western industrialized countries, the majority of whom spoke English as their mother tongue (Henrich et al., 2010b). In this study, however, we emphasize generalizability of the results from one language and culture to another. We compare four historically and culturally sufficiently different European samples with the aim of replicating findings across all of them.

## Methods

### Measures

All participants in this study completed either the Revised NEO Personality Inventory (NEO-PI-R; McCrae and Costa, 2010) or the NEO Personality Inventory-3 (NEO-PI-3; McCrae et al., 2005), which is a slightly modified version of the NEO-PI-R. Questions and wording in the NEO-PI-3 were altered to be more understandable to participants, to increase the accuracy of responses. Both versions use a five-point Likert scale ranging from “Strongly disagree” to “Strongly agree.” Like the original NEO-PI-R, the NEO-PI-3 has 240 items that measure 30 personality facets, which are grouped into the five FFM domains, such that each domain score is a composite of six facet scores. The NEO-PI-R/NEO-PI-3 has excellent psychometric properties across a wide range of languages and countries, including those which were included in the present study (De Fruyt et al., 2009).

### Participants

In total, there were 8230 participants in this study—4115 targets and 4115 knowledgeable informants from four different samples—Czech, Belgian (Flemish), Estonian, and German.

#### Czech sample

The Czech sample included 808 targets (329 men, 479 women) who were recruited in a series of studies (McCrae et al., 2004). They ranged in age from 14 to 83 years, with a mean age of 35.7 (*SD* = 14.2 years). Raters came from different study schemes, which were explained in our previous paper (Allik et al., 2010a). All participants used the Czech version of the NEO PI-R questionnaire (Hřebíčková, 2002).

#### Estonian sample

Participants for the present study came from the *Estonian Biobank* cohort, for which data were collected by the Estonian Genome Centre (EGC) at the University of Tartu (Leitsalu et al., 2014). In the Estonian sample, 2658 participants (1455 women and 1203 men) with a mean age of 46.0 years (*SD* = 17.3, ranging from 18 to 91 years) completed the self-report version of the Estonian NEO Personality Inventory-3. All 2658 participants nominated somebody who knew them well. Those who were nominated were asked to rate the personality traits of the specific target using the other-report version of the Estonian NEO-PI-3. Of the informants, 1845 were women (72.2%) and 723 were men. The mean age of informants was 42.6 (*SD* = 24.1) years.

#### Flemish sample

Flemish data were collected from 345 target participants (270 women and 75 men) who were psychology students at the Katholieke Universiteit Leuven and who, as a course requirement, rated their own personality with the Dutch version of the NEO PI-R (Hoekstra et al., 1996). They also recruited a well-acquainted person (*n* = 345; 190 women, 112 men, and 43 did not specify sex), either a relative or a friend, who rated their personality using the observer-report form of the same instrument. The mean age of targets was 18.4 (*SD* = 3.0) years. The mean age of external raters was 29.5 (*SD* = 13.7) years.

#### German sample

Participants were 304 students (169 women, 134 men, and 1 not reporting sex) at a German university, of whom only 3 studied psychology (Borkenau and Zaltauskas, 2009). Their mean age was 23.38 (*SD* = 2.68) years, ranging from 18 to 35 years. They received 45 euros for their participation and were recruited in 76 groups, each comprising four persons who all knew each other well. Each four-person group was split into two dyads and all participants described themselves and the other dyad member on several personality inventories including the German version of the NEO PI-R (Ostendorf and Angleitner, 2004).

The Czech, Flemish, and German samples have also been used in our previous studies (Allik et al., 2010a, 2012; Borkenau et al., 2013a).

### Normativeness and distinctiveness of personality profiles

Personality profiles reflect at least two different components—normativeness and distinctiveness (Cronbach, 1955; Furr, 2008; Borkenau and Zaltauskas, 2009). Profile normativeness is the degree to which a profile reflects an average profile—the similarity between an individual's profile of scores and a group's normative profile of scores. Profile distinctiveness is how much the individual is above or below average scores on each trait (Furr, 2008; Borkenau and Zaltauskas, 2009). Personality researchers are primarily interested in profile distinctiveness because uniqueness, not stereotypes, is their main concern. In addition it was demonstrated that self-enhancement and social desirability responding have very little influence on distinctive self-other agreement (Borkenau and Zaltauskas, 2009). Thus, in order to get distinctive profiles, all personality data were standardized traitwise by converting them into *z*-scores. In the Czech and Estonian data, standardization took place separately for four groups of participants (men and women, each divided into two groups: one younger and one older than 30 years). Here, the raw scores on all thirty personality facets were transformed so that a new score of zero represented the mean for each of the four separate groups and a difference of one from the mean indicated a difference of one standard deviation. Normalization was done separately for self-ratings and observer-ratings on the basis of the target age and sex. Besides eliminating practically all normativeness from profiles, this normalization also eliminates a large portion of variance caused either by age or sex differences. Since participants from the Belgian and German samples were, on average, relatively young, their data were standardized separately for sex only.

### Measures of profile agreement

Researchers have proposed various indices measuring the level of agreement between two personality profiles, including Cattell's Index, McCrae's Index, Intraclass correlation, and several others (McCrae, 1993, 2008; Furr, 2010). All of these various measures, however, are similar to the Pearson product moment correlation, because they estimate similarity of shapes as one of their components. It is not surprising that, usually, the Pearson *r* performs nearly as accurately as other measures of profile agreement (McCrae, 2008). In addition to its essential simplicity, it is important to note that the Pearson *r* does not depend on the specific measurement units used. If one of the profiles is affected by an elevation or differential elevation, then, as soon as these two profiles are linearly invariant to each other, the Pearson *r* regards them as identical. Based on these considerations, we used the Pearson *r* as a sufficiently fair representation of all other agreement indices.

## Results

### Profile agreement

We began by computing profile agreements *r*_*P*_ between self- and other-ratings for all 4115 participating dyads. Expectedly, when we used raw scores, mean profile correlations were relatively high (Table 1, first row). The mean value across all four samples was 0.57 (see the last column). When distinctiveness was separated from normativeness (by standardizing scores), profile agreement dropped by 0.16 points, on average. This suggests that distinctive agreement accounted for approximately 77, 73, 70, and 70% of overall profile agreement in the Czech, Estonian, Flemish, and German samples, respectively. However, individual profile correlations based on standardized scores were not always lower than those relying on raw scores. For 15.1% (Estonian sample) to 21.7% (Flemish sample) of all dyads, profile correlations based on standardized scores were higher, not lower, than the same correlation based on raw scores.

**Table 1:** **Mean profile correlations and Rank Consistency Indices for the four samples**.

**Mean profile correlations and consistency indices**	**Sample**				**Average**
	**Czech**	**Estonian**	**Flemish**	**German**	
Pearson r_P_ (raw data)	0.58	0.61	0.53	0.54	0.57
Pearson r_P_ standardized traitwise	0.45	0.45	0.37	0.37	0.41
Pearson r_P_ standardized trait- and personwise	0.46	0.46	0.37	0.37	0.42
*Rank Consistency Index* ρ_XY_	0.45	0.46	0.40	0.38	0.42

Analogously to standardizing scores trait- or column-wise, it is also possible to standardize scores person- or row-wise. Sometimes this type of standardization is called ipsatization, which is useful for eliminating peculiarities in a rater's style of responding (e.g., preference for one part of the response scale). As a result of ipsatization, the mean is zero, and the standard deviation is one, for each person. To standardize scores trait-wise and person-wise simultaneously, it may be necessary to iterate the standardization procedure along columns and rows several times to obtain column and row means and standard deviations sufficiently close to zero and one, respectively. The third row in Table 1 reports mean profile correlations based on double-normalized scores for our four samples. Obviously, these profile correlations are very similar to those in the previous row.

### Trait agreement

**Figure 1:**
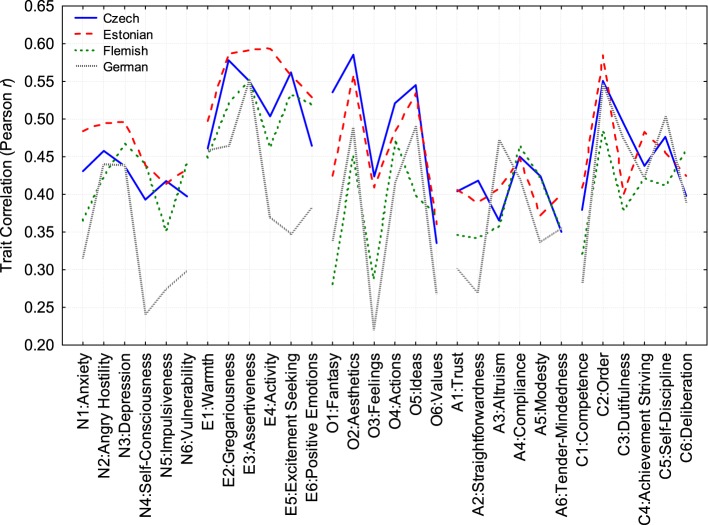
**Profiles of self-other trait correlations for the Czech, Estonian, Flemish, and German samples**.

Next, we computed self-other agreement for all NEO-PI facet scales. It should be noted that linear transformations of trait variables (adding, and multiplying by, a constant) do not affect their correlation. Figure 1 demonstrates the trait agreement values (Pearson *r*_*T*_) in our four samples for all 30 NEO-PI traits. The shape of these four profiles is similar: Their correlations across facets vary from 0.51 (Czech vs. Flemish) to 0.73 (Flemish vs. Estonian), suggesting that the pattern of agreement is generalizable from one language and/or culture to another. This indicates that individuals and their informants agree on some traits more than on others. For example, E3: Assertiveness is a trait on which it is easy to agree (average *r*_*T*_ = 0.56), whereas perceptions of self and informant on O6: Values agree least (average *r*_*T*_ = 0.32). The mean agreement across all traits varied from 0.32 (Germany) to 0.47 (Estonia), which is quite consistent with previously reported values (McCrae et al., 2004).

It is perhaps interesting to mention that it made little difference whether the Spearman rank-order correlation or the Pearson *r* was used. Self-other trait correlations computed on ranks were, on average, only slightly smaller than the Pearson correlations.

### Rank consistency index

Next, we decomposed self-other trait correlations into contributions by individual dyad, using the *RnkCI*, and computing ρ_XY_ -values for all 4115 dyads. As expected, the mean ρ_XY_-values (across all traits) were similar to the profile correlations of the same self-other dyads. The correlations between ρ_XY_ and *r_P_* were 0.75, 0.79, 0.51, and 0.67 for the Czech, Estonian, Flemish, and German samples, respectively. Although these correlations are relatively high, there is still a fair amount of freedom until a complete congruence. We therefore checked whether these correlations got higher by aggregating them over a sufficient number of occurrences (Epstein, 1979, 1980). We divided all samples into 10 approximately equal-sized groups on the basis on their profile agreement values *r_P_*. Figure 1 displays the mean values of profile correlation (r_P_) and the mean RnkCI *(*ρ_XY_) for these 10 groups.

Except for very few deviating points, the relationship is almost perfectly linear, suggesting that, if random noise is suppressed, profile correlations can be rather accurately predicted from mean differences in the ranking on personality traits. The correlation between the data points is 0.96 (*p* < 0.0001). If the individual (*X*) and the informant (*Y*) report sufficiently similar ranks on all, or at least many, personality traits, the two profiles are similar as well. The effects of aggregation show that most of the unexplained variance is indeed random because it is canceled out by the aggregation.

### Contribution of traits to profile agreement

We can also ask what each trait contributes to the self-informant profile correlation. Let us suppose that, in each individual profile, scores are replaced by their ranks: The NEO-PI facet scale scoring the highest receives a rank of one. The next highest score receives a rank of 2, and so on, until the lowest score receives a rank of 30. Self and informant scores are ranked separately. Now the difference between ranks for self- and other-ratings determines how much this particular trait contributes to the overall profile correlation. Traits which have identical or similar ranks in self and informant profiles contribute more strongly to profile correlation than those traits which have a large discrepancy between ranks. Thus, we can apply the same *Rank Consistency Index* or *RnkCI* ρ_XY_ to evaluate how much each trait contributes to profile correlations.

**Figure 2:**
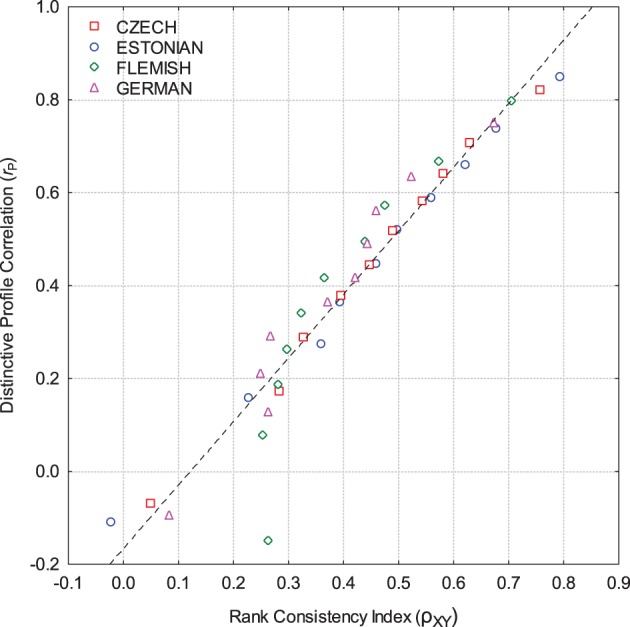
**Relationship between average profile correlation (*****r_P_*****) and average** ***RnkCI*** **(ρ_XY_), when samples were divided into 10 groups of equal size on the basis of their profile correlations**.

**Figure 3:**
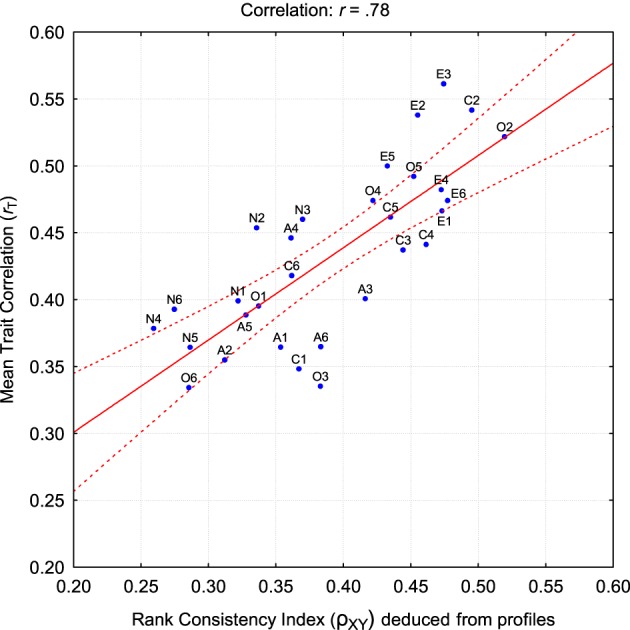
**The relationship between traitwise self-informant agreement and the contribution of the trait to the average profile correlation**.

For each individual dyad, we found 30 ρ_XY_-values, each showing how much a particular trait contributed to the profile correlation. After that, we computed the mean consistency value ρ_XY_f for each trait by averaging scores across all *N* self-informant pairs in each of our four samples. This averaged *RnkCI* indicates how much this particular personality trait contributes to the profile correlation averaged across all participants in that sample. This allows us to check if the contributions of that trait to profile agreement are related to the self-other agreement *r*_*T*_ for that trait (as reported in Figure 2). The correlation between ρ_XY_ and *r_T_* was 0.79, 0.80, 0.54, and 0.87 for Czech, Estonian, Flemish, and German samples, respectively. For greater clarity, we illustrate the relationship between ρ_XY_ and *r_T_* for the combined sample of 4115 targets in Figure 3.

This figure shows that traits showing higher self-other agreement are the same traits as those contributing significantly to the self-other profile correlations. The average correlation between contributions to profile agreement (ρ_XY_) and *r_T_* was sufficiently high, *r* = 0.78, *p* < 0.0001. For example, E3: Assertiveness, C2: Order, and O2: Aesthetics are high on both axes. At the same time, N4: Self-Contentiousness, O6: Ideas, and A2: Straightforwardness contributed relatively modestly to both measures of agreement. Nevertheless, it is useful to note that both values are in a relatively narrow corridor, somewhere around the value 0.40. This means that relatively good agreement is achievable on all traits without clear distinctions between dimensions that are more or less judgeable.

## Discussion

As a result of this study, it is perhaps time to say goodbye to two still widely held misconceptions. One of them is the belief that trait correlations (or trait-by-trait analysis in general) do not provide information on the agreement between individual judge pairs. Similarly, it was considered self-evident that profile analysis does not permit assessment of the relative contributions of traits to overall accuracy (Bernieri et al., 1994, p. 370). Asendorpf (1990) proposed, already 25 years ago, a simple idea for decomposing the overall Pearson correlation into the individual contributions of each of the pairs of scores from which a correlation is computed. The proposed consistency index *I_XY_* implements the intuitively transparent idea that those pairs which occupy approximately the same ranks on the trait continuum contribute strongly to the overall correlation, whereas those members of the dyads that occupy very different ranking positions contribute less, or negatively, to the overall trait correlation. To implement this basic intuition more literally and to overcome the limitations of the Asendorpf index, we proposed the *Rank Consistency Index* ρ_XY_ or RnkCI, which is proportional to the squared difference between the ranks of self-ratings and of informant ratings on a given trait. More precisely, the mean value of individual indices ρ_XY_ is equal to the overall Spearman ρ being computed on the same ranks. Thus, the RnkCI indicates how much a particular judge pair contributed to that trait correlation. Similarly, we can decompose self-other profile correlations into the relative contributions of each trait. It is surprising that these mathematically elementary ideas were, to the best of our knowledge, never applied to self-other agreement.

We demonstrated, for the first time, that there are two alternative ways to determine which traits are perceived more consensually by judges. One usual way is to compute self-other correlations trait by trait. From such analyses, we learned that those traits that were perceived more consensually in one culture were also perceived more consensually in the other samples under study. For some reason, E3: Assertiveness, C2: Order, and O2: Aesthetics showed high self-other agreement in all four samples. Although sufficient agreement was achieved on all 30 personality traits being studied, self-other agreement was lower for self-consciousness, openness to new ideas, and straightforwardness. However, when inspecting the traits which contributed more to individual profile correlations the same pattern emerged. Aggregating consistency indices across all dyads, it turned out that the rank differences were smaller for those traits on which higher self-other agreement was obtained. In turn, rank consistency was relatively small (ranks were different) for assertiveness, order, and openness to aesthetics, on which trait agreement was harder to obtain.

Similarly, there are two alternative approaches for deciding which of all participating dyads agrees more in judgment of personality. Computing correlations between profiles of self- and other-ratings reveals dyads which reach high, intermediate, or low agreement. An alternative way to find dyads excelling in self-other agreement is to take differences in the ranks that members of the same dyad occupy on all personality traits into account. As we demonstrated, these two alternative ways of characterizing dyad agreement converge strongly. The correlation between profile agreement *r_P_* and rank consistency ρ_XY_ was in the range of 0.51 (Flemish sample) to 0.79 (Estonian sample). One can complain, of course, that correlations such as 0.51 are not very impressive, leaving plenty of room for psychologically meaningful differences between these two ways of characterizing agreement. This argument appears to carry even more weight knowing that the use of the original Asendorpf index *I_XY_*, instead of ρ_XY_, led to similar, but lower, convergence. However, the aggregation test we applied demonstrated that the main problem was that the dyad was too small as a unit of analysis to obtain reliable findings. If the unit of analysis comprised a sufficient number of occurrences, these two alternative ways of computing within-dyad agreement converged very nicely, leaving approximately 8% of variance unexplained. Not denying that even 8% of variance can have a psychologically meaningful interpretation, this is still compelling evidence that these two alternative methods converge substantially.

We need to clarify one potential source of controversy. In one of our previous studies, we claimed that agreement only moderately generalizes from one personality trait to another (Allik et al., 2010b): A reliable judge of one's own or a friend's openness is not necessarily a good judge of, for example, conscientiousness. One consequence of this lack of generalizability is that there may be no good judges of personality because, on the basis of how well you judge openness, it is not possible to say how well you judge other personality traits (for the oppoite vew, see Funder, 1997, 1999). In addition to this puzzle, our previous results seem to contradict the present findings that trait agreement, when decomposed into the contributions of individual pairs of raters, correlated substantially with the coefficients of profile agreement. A crucial difference between our current and previous studies is in the level of analysis. Instead of all 30 facets, in the previous study, we were interested in domain scores only, as well as computing Asendorpf's index for them. This also means that facets loading on the same dimension are correlated more strongly with one another than with facets which contribute to other dimensions. Since in our previous study (Allik et al., 2010b) we observed generalizability across dimensions, we missed generalizability between facets measuring the same broad traits. Thus, agreement is definitely generalizable across facets of the same trait dimension. If someone is, for example, an accurate judge of his or her own or a friend's anxiety, hostility, or depression, he or she is also most likely good at rating impulsiveness, vulnerability, or self-consciousness, being all different facets of neuroticism.

Even after establishing substantial convergence between two alternative ways of estimating agreement, it remains to be explained why some factors (e.g., length of cohabitation) can affect, for example, trait agreement but not profile agreement (Bernieri et al., 1994). Undoubtedly, Michael Furr was one of first to point out the fact that, despite the meaningfulness of both approaches, they emerge from precisely the same data (Furr, 2009, p. 203). He was, however, probably mistaken when he claimed that the connection between trait-centered and person-centered approaches cannot be drawn clearly on a correlational metric (Furr, 2009, p. 203). As we demonstrate in the Appendix A in Supplementary Material, the connection between trait and profile correlations becomes obvious when the Pearson correlations are expressed as the means of the products of the standard scores. Expressing the profile (*r_P_*) and trait (*r_T_*) correlation through the product of the standard scores makes it evident that these two formulas share a common term—the product of the self- and other-rated scores. This means that there is a direct link between these two forms of agreement, which makes them dependent on each other. Even if there is no strict one-to-one relationship, there has to be a positive association between these two forms of correlations.

Personality psychologists are rarely interested in the normativeness component in personality profiles—how much a given person is similar to an average or prototypic person of the group to which he or she belongs. Personality researchers are usually interested in the distinctiveness component of judgments, which show how much the individual is above or below average on each trait. One of the most common ways to measure this is to standardize data by subtracting the sample mean from each score and dividing the result by the standard deviation of that sample. This yields a standardized variable with a zero mean and unit variance. If we compute the Pearson *r* between two standardized variables, then the result is simply equal to the mean product of these standardized scores. This leads to the conclusion that, if personality data are double standardized (all means of columns and rows are equal to zero with a unit standard deviation), then the mean profile correlation r_P_ (averaged across all pairs of judges) is equal to the mean trait correlation r_T_ (averaged across all traits). This also provides an explanation as to why some factors can affect trait agreement but not profile agreement, or vice versa. This can happen only when traits differ in their levels of elevation and/or their variances. Another possibility is that individual profiles have different levels of elevation and/or different scatter patterns of scores within profiles. Usually, these factors are considered artifacts and controlled by standardization. For example, differential scatter of profiles is typically perceived as being produced by a rater's style of responding, not the judged traits themselves. To get rid of differences in response styles, it is recommended to use ipsatized profiles. As a result, differences between variable-centered and person-centered approaches almost vanish.

This conclusion has consequences for the long-lasting debate about the merits of person-by-person and trait-by-trait approaches to personality (Magnusson and Torestäd, 1993; Pelham, 1993; Bernieri et al., 1994; Asendorpf, 2000; Bergman and Trost, 2006; Furr, 2009). Our position is more in line with those authors who do not think that it is important whether we start our analysis from traits or from the individual (Asendorpf, 2000; Furr, 2009). It is also not the case, as we have already mentioned, that trait-by-trait analysis does not provide information regarding the accuracy of a particular judge, and that profile analysis does not permit assessment of the relative contributions of traits to overall accuracy. Because trait and profile agreement are related, studying traits has substantial implications for the individuals being studied. At the same time, focusing on individuals has substantial implications for traits, for example, whether they are perceived consensually.

When McCrae and Costa (1997) proposed the bold hypothesis that the pattern of covariation among personality traits may be a human universal, they had to rely on only six translations of the NEO Personality Inventory (NEO-PI-R), that is, into German, Portuguese, Hebrew, Chinese, Korean, and Japanese. Nevertheless, data from these highly diverse cultures with languages from five distinct language families were persuasive enough to suggest that the observed pattern of covariation among personality traits would be very similar when new cultures and languages were subjected to critical examination (McCrae and Allik, 2002). It turned out, however, that the pattern of covariation is easily generalizable not only across cultures and languages, but many other properties as well (Allik et al., 2013). For example, in most cultures studied, men vary more than women in personality (Borkenau et al., 2013a,b). On the other hand, there is a cross-culturally replicable pattern of differences between internal (self) and external (informant) perspectives on the Big Five personality traits (Allik et al., 2010a). This study extends that list, demonstrating that there is a replicable pattern of self-other agreement, which generalizes across four studies, cultures, and languages.

Average self-other agreement was slightly higher in the Estonian and Czech samples (0.46) and more modest in the Flemish and German samples (0.36–0.37). It is, of course, possible that this division reflects some cultural or even historical differences. It is, however, more likely that these differences in self-other agreement reflect differences in the demographic composition of these samples. For instance, the Czech and Estonian samples were not only the two largest but also the two oldest samples. The Flemish and German participants were mainly young students. Students are more homogenous, not only in their age composition, but also in their level of education. Personality differences may be smaller in such a sample of students than in populations with a wider range of demographic characteristics. Nevertheless, it is also possible that students were less motivated in doing this task and, as a result, provided more random responses. Alternatively, fellow students may not have known their targets as well as the relatives or partners in the adult samples. These are, of course, pure speculations. More studies are needed to understand how demographic variables, including culture, influence self-other agreement on personality traits.

In general, these relatively small variations between samples do not obscure the main conclusions. If we compute self-other agreement on some personality traits then we possess all information we need about the individuals who are the targets of this judgment. Analogously, we can always decompose agreement between two personality profiles into the individual contributions of traits from which these profiles are assembled. The period of uncertainty that two principal ways of computing the self-other agreement—trait and profile agreement—sometimes reveal similar, and sometimes dissimilar, findings is over. We know exactly when grand means of the trait and profile agreement, r_T_ and r_P_, converge into an identical value. The elevation and scatter of some traits and profiles relative to other traits and profiles are the only reason why the mean trait correlation r_T_ and the mean profile r_P_ are not always equal.

## Author notes

Preparation of this manuscript was supported by the University of Tartu (SP1GVARENG) and by institutional research funding (IUT2-13) from the Estonian Ministry of Education and Science. AR was supported by a grant from the Netherlands Institute for Advanced Study (NIAS) during the preparation of this article. The authors are grateful to Andres Metspalu and Tõnu Esko for access to the Estonian data. We also thank Delaney Michael Skerrett for his helpful comments on earlier drafts of this article.

### Conflict of interest statement

The authors declare that the research was conducted in the absence of any commercial or financial relationships that could be construed as a potential conflict of interest.
