# Supplementary material for: How are personality trait and profile agreement related?
Source: Front Psychol. 2015 Jun 9;6:785. doi: 10.3389/fpsyg.2015.00785 (PMC4460800; doi:10.3389/fpsyg.2015.00785)
Supplement: Supplementary file 1 [file Presentation1.PDF]

How are Personality Trait and Profile Agreement Related?

Appendix A

Let *i* denote individuals (*i* =1, 2, ..., *N*) and *j* denote traits (*j* =1, 2, …, *K*), and *x* and *y* the scores of self- and other-rated personality traits, respectively. Thus, we can represent self-informant rating data as two corresponding *K* × *N* matrices, populated with the self- and other-rating scores x_{ij} and y_{ij}, respectively (see Table A1).

Table A1

Schematic View of How Self-Other Rating Data Can be Represented as Two Conjoint Matrices

Dyads	Traits *j* = 1, 2, … , *K*													
i=1,2, …*N*	1		2		…	j		…	*K*		Mean		StdDev	
1	x_{11}	y_{11}	x_{12}	y_{12}	⋯	x_{1j}	y_{1j}	⋯	x_{1K}	y_{1K}	$\bar{x}_{1\cdot}$	$\bar{y}_{1\cdot}$	s^2_{1X}	s^2_{1Y}
2	x_{21}	y_{21}	x_{22}	y_{22}	⋯	x_{2j}	y_{2j}	⋯	x_{2K}	y_{2K}	$\bar{x}_{2\cdot}$	$\bar{y}_{1\cdot}$	s^2_{2X}	s^2_{2Y}
…	...		…		…	…		…	…		…		…	
i	x_{i1}	y_{11}	x_{11}	y_{11}		x_{ij}	y_{ij}		x_{iK}	y_{iK}	$\bar{x}_{i\cdot}$	$\bar{y}_{i\cdot}$	s^2_{iX}	s^2_{iY}
…	…		…		…	…		…	…		…		…	
N	x_{N1}	y_{N1}	x_{N2}	y_{N2}	⋯	x_{Nj}	y_{Nj}	⋯	x_{NK}	y_{NK}	$\bar{x}_{N\cdot}$	$\bar{y}_{N\cdot}$	s^2_{NX}	s^2_{NY}
Mean	$\bar{x}_{.1}$	$\bar{y}_{.1}$	$\bar{x}_{.2}$	$\bar{y}_{.2}$	⋯	$\bar{x}_{.j}$	$\bar{y}_{.j}$	⋯	$\bar{x}_{.K}$	$\bar{y}_{.K}$				
StdDev	s^2_{X1}	s^2_{Y1}	s^2_{X2}	s^2_{Y2}	⋯	s^2_{Xj}	s^2_{Yj}	⋯	s^2_{XK}	s^2_{YK}				

For each row (pairs of judges) and column (traits), we can compute the means and standard deviations of self-ratings and the other-ratings. For example, across rows, we can find the mean $\bar{x}_{i\cdot}$ and the uncorrected variance of the sample s_{ix}^2 of self-ratings for the *i*-th dyad:

$$\bar{x}_{i\cdot} = \frac{1}{K}\sum_{j=1}^{K} x_{ij}.$$

$$s_{iX}^2 = \frac{1}{K}\sum_{j=1}^{K} (x_{ij} - \bar{x}_{i\cdot})^2 .$$

Note that we are not using the correction *K*–1, which is necessary when the variance of the entire population is estimated. Replacing x_{ij} with y_{ij}, one can find the mean $\bar{y}_{i\cdot}$ and uncorrected variance s_{ix}^2 for other-ratings as well.

Analogously, we can find means ($\bar{x}_{\cdot j}$ and $\bar{y}_{\cdot j}$) and variances (s_{Xj}^2 and S_{Yj}) going down the columns for each *j*-th trait and for self- and other-ratings, respectively:

$$\bar{x}_{\cdot j} = \frac{1}{N}\sum_{i=1}^{N} x_{ij}.$$

$$s_{Xj}^2 = \frac{1}{N}\sum_{i=1}^{N} (x_{ij} - \bar{x}_{\cdot j})^2.$$

Formulas are given only for the self-ratings *X*, but it is easy to devise similar formulas for $\bar{y}_{\cdot j}$ and s_{Yj}^2.

The Pearson correlation r_{Pi} between self and informant profiles for a given dyad *i* can be found by the following formula:

$$r_{Pi} = \frac{1}{K}\sum_{j=1}^{K}\left(\frac{x_{ij} - \bar{x}_{\cdot j}}{\sqrt{s_{iX}^2}}\right)\left(\frac{y_{ij} - \bar{y}_{\cdot j}}{\sqrt{s_{iY}^2}}\right).$$

Please note that the correlation coefficient r_{Pi} is expressed here as the mean of the products of the standard scores. Again, we need to drop the correction *K*–1 to make the different formulas compatible. The use of the correction is of theoretical interest only because it barely affects computational results, except if *N* or *K* is extremely small.

Summing r_{Pi} across all *N* dyads and dividing by their total number, we can find the mean profile correlation $\bar{r}_T$ across all individuals *N*:

$$\bar{r}_P = \frac{1}{N}\sum_{i=1}^{N} r_{Pi}$$

Analogously, we can find the trait correlation coefficient r_{Tj} for each out of *K* traits:

$$r_{Tj} = \frac{1}{N}\sum_{i=1}^{N}\left(\frac{x_{ij} - \bar{x}.j}{\sqrt{s_{Xj}^2}}\right)\left(\frac{y_{ij} - \bar{x}._j}{\sqrt{s_{Yj}^2}}\right).$$

The mean value across all *K* traits gives the mean trait correlation $\bar{r}_T$:

$$\bar{r}_T = \frac{1}{K}\sum_{j=1}^{K} r_{Tj}$$

The two correlation coefficients, r_{Pi} and r_{Tj}, are directly proportional to the product $x_{\mathrm{ij}} \times \underline{y}_{\mathrm{ij}}$ of the self-rating and other-rating standard scores: the more they deviate from their respective mean values in the same direction, the larger both correlation coefficients become.

If data in Table A1 are standardized across traits (columns) in a such way that all means are zero and all standard deviations one, then the mean trait correlation $\bar{r}_T$ reduces to the level of the mean product $z_{ij}^X \times z_{ij}^Y$ (*X* and *Y* are used here as indices of self- and other-reports, not exponents) of the standardized scores of self-ratings (*x*) and other-ratings (*y*):

$$\bar{r}_T = \frac{1}{KN}\sum_{j=1}^{K}\sum_{i=1}^{N} z_{ij}^X z_{ij}^Y$$

Similarly, one can standardize data across individual profiles (rows). Usually, this type of standardization is called ipsatization, the purpose of which is to eliminate idiosyncrasies in a rater's style of responding. Ipsatized scores are calculated based on the mean and standard deviation for each rater across traits, implying that the mean and standard deviation for each rater are zero and one, respectively. It may be necessary to iterate normalization several times to obtain a data matrix in which all column (trait) and row (rater) means and standard deviations are sufficiently close to zero and one, respectively. The mean trait correlation $\bar{r}_P$ calculated based on double-normalized *z*-scores is:

$$\bar{r}_P = \frac{1}{NK} \sum_{i=1}^{N} \sum_{j=1}^{K} z_{ij}^X z_{ij}^Y$$

In other words, the formulas for the mean trait $\bar{r}_T$ and the mean profile $\bar{r}_P$ correlations are identical, provided that data are double-standardized across columns (traits) and rows (profiles). Thus, after double standardization, there is only one overall measure of mean self-other agreement.

Expressing correlations as products of standard scores also explains why the mean trait correlation $\bar{r}_T$ can be different from the mean profile $\bar{r}_P$. The reason is that the means and standard deviations of the columns (traits) and rows (profiles) are not equal. In other words, the elevation and scatter pattern of some traits and profiles relative to other traits and profiles are the reason why the mean trait correlation $\bar{r}_T$ and the mean profile $\bar{r}_P$ are not equal.

Appendix B

To demonstrate that double standardization across columns (traits) and rows (profiles) leads to (nearly) identical the mean trait $\bar{r}_T$ and the mean profile $\bar{r}_P$ correlations we compiled a random sample of data (Table B1). In ten dyads (d_i) each target was judged by him- or herself in addition to a knowledgeable observer on the Big Five personality scale measuring Neuroticism, Extraversion, Openness, Agreeableness, and Conscientiousness. Forty-eight items with a Likert 5-point response scale represented each dimension.

Table B1

A Demo Set of the Sum Scores of the Personality Self- and other-ratings on the Big Five Personality Traits Made in Ten Dyads

	N		E		O		A		C		
Dyad	Self	Other	Self	Other	Self	Other	Self	Other	Self	Other	r_P
d_1	67	65	95	110	107	104	126	123	119	127	*0.94*
d_2	113	119	99	103	102	100	94	53	132	137	*0.85*
d_3	73	85	172	139	124	118	54	80	87	95	*1.00*
d_4	71	95	91	92	81	95	118	108	142	142	*0.91*
d_5	121	122	79	72	105	88	92	109	128	152	*0.86*
d_6	100	83	80	119	86	109	98	83	127	77	*-0.85*
d_7	120	108	103	129	83	87	113	110	105	133	*0.43*
d_8	80	67	119	112	116	97	122	122	151	163	*0.97*
d_9	38	44	142	127	119	117	121	152	147	148	*0.93*
d_{10}	102	123	107	74	78	43	109	79	111	126	*0.68*
r_T	*0.87*		*0.61*		*0.65*		*0.61*		*0.60*		

Note: d_i = dyad number; N = Neuroticism; E = Extraversion; O = Openness; A = Agreeableness; C = Conscientiousness; r_T = Trait agreement; r_P = Profile agreement.

The last column (r_P) demonstrates the profile correlation. Except dyad d_6 profile correlations are remarkably high. The mean profile correlation is $\bar{r}_P$ = .56. The last row demonstrates the trait correlation r_T for each five personality trait. The mean trait correlation $\bar{r}_T$ = .67 is higher than the mean trait correlation. For example, in the Estonian sample (*N*=2,658) used in this study the mean profile correlation $\bar{r}_P$ = .66 and the mean trait correlation $\bar{r}_T$ = .56.

In Table B2 the same set of data is shown after repeated double normalization. The sum scores were first normalized along columns and then along rows. For a convergence, standardization was repeated several times until all means across columns and rows were close enough to zero with approximately unit standard deviation.

Table B2

The Dataset Shown in Table B1 after Repeated Double Normalizations of the Sum Scores

	N		E		O		A		C		
Dyad	Self	Other	Self	Other	Self	Other	Self	Other	Self	Other	r_P
d_1	-0.74	-1.25	-0.54	0.01	0.80	0.67	1.46	1.24	-0.95	-0.67	*0.92*
d_2	1.00	0.76	-0.51	0.15	0.53	0.52	-1.63	-1.74	0.57	0.31	*0.94*
d_3	-0.14	-0.01	1.43	0.98	0.72	1.02	-1.03	-0.88	-0.96	-1.11	*0.96*
d_4	-0.45	0.27	-0.33	-1.63	-1.37	-0.12	0.96	0.44	1.19	1.03	*0.52*
d_5	0.97	0.94	-1.21	-1.49	1.03	-0.42	-1.00	0.14	0.17	0.83	*0.54*
d_6	0.96	0.12	-1.25	0.72	-0.91	1.07	0.07	-0.45	1.08	-1.46	*-0.79*
d_7	0.84	0.57	0.67	1.27	-0.88	-1.39	0.78	-0.08	-1.42	-0.36	*0.70*
d_8	-1.41	-1.26	-0.51	-0.16	0.83	-0.49	-0.07	0.54	1.19	1.36	*0.73*
d_9	-1.55	-1.54	1.22	0.18	0.65	0.46	-0.28	1.15	0.02	-0.24	*0.62*
d_{10}	0.52	1.24	1.03	-0.25	-1.39	-1.45	0.73	-0.07	-0.89	0.53	*0.44*
r_T	*0.88*		*0.43*		*0.42*		*0.66*		*0.39*		

Note: The same as in Table B1.

After double-normalization, some of the agreement values, r_P or r_T, decreased but some values increased. As predicted by Appendix A, the mean profile and trait agreement became approximately identical, $\bar{r}_P \approx \bar{r}_T \approx .55$.

Please note that the mean value of the products of standard self and other rated scores within each of ten (dyads) by five (traits) cells is equivalent to the both mean profile and trait agreement.
